# The ability to value: An additional criterion for decision‐making capacity

**DOI:** 10.1111/bioe.13387

**Published:** 2024-12-16

**Authors:** Lauren Harcarik, Scott Y. H. Kim, Joseph Millum

**Affiliations:** ^1^ School of Social Work University of Maryland College Park Maryland USA; ^2^ Department of Bioethics National Institutes of Health Bethesda Maryland USA; ^3^ Department of Philosophy University of St. Andrews St Andrews UK

**Keywords:** autonomy, capacity assessment, competence, decision‐making capacity

## Abstract

In the United States, the dominant model of decision‐making capacity (DMC) is the “four abilities model,” which judges DMC according to four criteria: understanding, appreciation, reasoning, and communicating a choice. Some critics argue that this model is “too cognitive” because it ignores the role of emotions and values in decision‐making. But so far there is no consensus about how to incorporate such factors into a model of DMC while still ensuring that patients with unusual or socially disapproved values still have their autonomous decisions respected. In this paper, we aim to give an account of the role of values in decision‐making which can answer some of the lingering questions about capacity. In the current literature, defenders of the inclusion of values in DMC tend to propose solutions which focus on the distorted or incoherent attributes of the values themselves. We argue that shifting the focus onto valuing as an ability is a better solution and that a complete picture of capacity includes understanding, appreciation, reasoning, communicating a choice, *and* the ability to value. On the basis of a conceptual analysis of the necessary conditions for autonomous decision‐making, we derive a conception of the ability to value. On our account, the ability to value has four components: the possession of values, the ability to access those values, the ability to engage in practical reasoning with one's values, and the ability to act on the result of that reasoning. We describe the positive components of the ability to value, some indicators of impairment, and some implications of our account.

## 1 INTRODUCTION

Adult patients with decision‐making capacity (DMC) have the right to accept or decline medical interventions according to their own judgment. In cases where DMC is lacking, decisions should be made by another person on that patient's behalf. Clinicians are often charged with assessing whether the patient has DMC when there is doubt. It is therefore vitally important that when clinicians assess DMC, they are measuring the *relevant abilities*—that is, the abilities that ground the right to make one's own decisions.

The standard models of DMC in the United States and the United Kingdom have been criticized for focusing too narrowly on cognitive skills and ignoring the emotional and evaluative elements of capacity.^1^ Consider a young, severely depressed person who says they have no hope for their future, and therefore refuses life‐saving treatment for an acute illness. The patient might meet all the standard criteria for DMC: they comprehend the facts about the proposed treatment (that it can be lifesaving), say they could die without it, seem to be able to reason about their situation (e.g., by articulating the consequences of different choices), and communicate a preference (that they prefer to do nothing). Nonetheless, their doctor might reasonably still doubt their DMC. What could be going wrong? We propose that the problem is sometimes not with the patient's ability to comprehend or process information, but with impairment in the abilities that allow them to value and act on the basis of their values.

In this paper, we consider whether and how current models of DMC should be augmented with assessments of patients’ abilities to use values in their decision‐making. We begin by deriving a conception of the *ability to value* from the conditions for autonomous decision‐making. According to this conception, the ability to value has four components: possession of values, access to one's values, practical reasoning with one's values, and the ability to act on the result of that reasoning. Reliable and observable indicators of impairment are necessary for clinicians to assess DMC, so we show how our account can provide clinicians and assessors with a framework for detecting impairments to the ability to value. We argue that a determination of lack of DMC on the grounds that a patient's ability to value is impaired should depend on evidence of both substantial impairment to their ability to value *and* evidence of pathology related to the impairment. We then apply our model to some tough cases. We conclude with a discussion of the potential objections to and limitations of using the ability to value criterion in DMC assessment.

## 2 THE STANDARD PICTURE AND ITS CRITICS

In the United States, the “four abilities model” of DMC is widely accepted. According to this model, patients have DMC if they have: (1) comprehension of the relevant information regarding their medical decision (*understanding*), (2) the ability to apply that information to their specific situation (*appreciation*), (3) abilities related to rational manipulation of the relevant information (*reasoning*), and (4) the ability to express a choice (*communication of choice*).^2^ Similar (overlapping but not identical) criteria are used in England and Wales, where the Mental Capacity Act (MCA) states that a person lacks capacity if the person is unable: (1) to understand relevant information, (2) to retain the information, (3) to use or weigh the information, or (4) to communicate a decision.^3^

A number of scholars have criticized the standard picture by pointing to cases where it struggles to capture apparent deficits in DMC that seem connected to patient values or emotions. For example, Tan et. al describe concerns about capacity in patients with anorexia nervosa as related to “pathological values.”^4^ As a result of their anorexia, patients report, “alterations of values, devaluation of other aspects of life, unusual values, and integration of anorexia nervosa into personal identity.”^5^ The same group raises further concerns with regard to how affective states in anorexia patients may inhibit their decision‐making abilities: “The power of the emotions in directing the person's decisions calls into serious doubt the validity of the person's refusal of treatment […].”^6^ Halpern raises a slightly different concern about the impact of emotions on capacity in her discussion of Ms. G—a patient who refused dialysis when her husband told her he was leaving her. Halpern argues that patients like Ms. G experience “unrelenting” emotional states which effectively concretize certain beliefs, rendering them unresponsive to outside evidence.^7^ When a patient experiences a concretized emotion‐belief complex, they are unable to deliberate about their decision, and DMC is impaired.

Despite the widespread concern that the standard picture does not properly incorporate patient values or emotions, no general framework for how values and emotions fit into DMC has been developed and defended. Indeed, after a comprehensive review of the literature on this topic, Hermann et al. express skepticism about the possibility of such a conception. They emphasize the case‐specificity of judgments about capacity that derive from considerations of patients’ emotions or values, the different interpretations that can be put on the same events, and the “conclusive argument” that emotions cannot be measured or operationalized to a reliable enough degree for assessment.^8^

One possible response to Hermann et al.'s skepticism is to radically rethink what underlies judgments of DMC. In a recent paper, Jennifer Hawkins attempts this by reconceptualizing competence along welfarist lines.^9^ She argues that we should supplement the four abilities model with a further prudential condition. Someone can be judged to lack capacity when there is good evidence they are making a serious prudential mistake *and* they have a condition that “is known to make those who have it more likely than ordinary to make prudential mistakes.”^10^ We believe that the problem can be solved without reconceptualizing the basis for DMC. Instead, we think that the traditional conception of competence as reflecting autonomous decision‐making can be expanded to include the ability to value; this ability can be operationalized sufficiently to be incorporated into assessments of DMC.

## 3 THE ABILITY TO VALUE

The ultimate ethical ground for ascribing a right to make medical decisions to all and only those with DMC is *respect for autonomy*. Provided that they do not harm or wrong others, autonomous individuals have the right to make decisions for themselves, even if those decisions are contrary to their interests. Being autonomous, then, is synonymous with having DMC. What is it to be autonomous? In the words of the Belmont Report, “An autonomous person is an individual capable of deliberation about personal goals and of acting under the direction of such deliberation.”^11^ Deliberation requires that one be able to understand information relevant to a decision, appreciate its relevance to one's situation, and reason with it. Action, in the context of medical decision‐making, requires that one communicates the results of deliberation. An autonomous medical decision, then, requires that a person must have the four abilities included in the standard picture of DMC. But deliberation can go nowhere without values. It is one's values—or, for the Belmont Report, one's “personal goals”—that tell one which outcomes matter, which actions are permissible, and so on. They are an integral part of practical reasoning—reasoning about what one *should* do.

Moreover, it is commonly thought that the reason others should respect the decisions of autonomous individuals is tied to the decision‐maker's possession and use of values.^12^ Making decisions on the basis of one's values is an important component of human flourishing; the ability to do so grounds a claim against others not to interfere with one's decision‐making. DMC therefore requires that an individual have what we label the *ability to value*.

The idea that autonomous action is a matter of making decisions in the light of one's values is common in the philosophical and bioethics literature.^13^ To spell out what this means in the context of assessing DMC requires articulating what a value is and what the component parts of the ability to value are. We do this by way of an example of decision‐making by a competent individual.

It's flu season, and I have to decide whether to get a flu shot. My options are either get the flu shot or not. Assume that I understand the facts about the flu and the vaccine, including how they apply to my situation. I know about the side effects and about how likely I am to experience them (based on the data and my own past experience of influenza vaccination). I know how much time this would take from my day. I know what it is like to have the flu. Plus, I have a rough idea about how the vaccine reduces the risks of getting the flu and how herd immunity protects people more vulnerable than myself. All this information is not enough for me to make a decision: something also has to *matter* to me. If nothing matters, then I won't be motivated to make one choice over the other. If some things do matter, then they can provide reasons for me to choose on the basis of how important I think they are.

I think that my health is important and I don't want to be inconvenienced by having to go to the clinic. I also value the health of those in my household and not contributing to the spread of the flu in my community. In making my decision, I will weigh my values against each other to determine which is most important and consider how, given the facts, my values are best expressed. Here, reasoning is relatively easy, since I am confident that the health of my household and community is more important than my short‐term convenience. On the basis of this reasoning, I decide to get the flu shot, and then I indeed go get it.

The above example suggests that the process of exercising the ability to value in making a decision can be decomposed into four components: the possession of values, the ability to access one's values, the ability to engage in practical reasoning with one's values, and the ability to act on the result of that reasoning. It should be noted that, depending on the circumstances, arriving at a decision can operate automatically or in the background: explicit deliberation might not take place. Nonetheless, if someone possesses the ability to value it should be possible to reconstruct the basis for their decision, including the underlying values.

The first component is the possession of relevant values. What are values? Values are what we express when we make judgments about good and bad, right and wrong, important and trivial, should and should not. To use such a value term is to indicate our view that something matters and that it matters in some sense independent of us. In this respect, values are different than desires. To value something is to think that it matters, whether or not I want it. I can want something, while believing that it is unimportant or even while wishing that I did not want it; not so with what I value.^14^ This feature of values makes them a source of reasons. For example, if I value my health, that gives me a reason to do things that are healthful. It is also something that I can cite in an explanation of *why* I made a decision—not just as a causal explanation but as a justification. For example, I chose to get the vaccine *because* I value my health. As sources of reasons, values are also typically stable. Our values may change over time, but they usually change gradually and in a piecemeal fashion.

The second component of our model is the ability to access one's values. In order to make a decision in the light of one's values, it is necessary not only to possess values but to use them, and that requires accessing the values. For example, in my decision about the flu shot, I access my values when I consider what can be said in favor of each option. This access need not be explicit: many times our values guide our decisions without active reflection and awareness.

The third component of the ability to value is the ability to conduct practical reasoning—that is, to apply one's values to a specific decision. This includes processes like comparing and prioritizing values. It is especially important in decisions with conflicting values. We call this *practical* reasoning since it deals with the specific reasoning process involved in coming to a decision about what to do. It is distinct from merely reasoning with relevant facts, even about oneself and one's situation. (In this sense, we think the MCA's criterion of “use or weigh” is a welcome development; that it is difficult to define and apply,^15^ we think, merely tracks the difficult terrain of talking about “ability to value” in decision‐making in general). In the flu shot example, I weigh my preference for convenience against the value I place on my personal health and the health of my community, and I prioritize the latter. This is an exercise of practical reasoning.

The fourth component of the ability to value is the ability to act on the basis of one's values—for example, by going to the clinic or giving consent to a procedure. This component has to do with being able to follow through on the conclusion of practical reasoning. A decision which does not motivate action is not a complete decision. If I say I'm going to get the flu shot, am not prevented from getting it by any external constraints, but then take no steps toward getting it, my values have not really motivated a decision. Of course, there is no guarantee that someone will be successful in their action: external impediments to freedom can prevent them from achieving what they attempt. The ability to act, as a component of DMC, is about whether someone is motivated by the results of their practical reasoning, not about whether they complete what they attempt.

## 4 IMPAIRMENTS OF THE ABILITY TO VALUE

Impairments to the ability to value can manifest at any stage of the decision‐making process and within any of the four components of the ability to value. Multiple impairments can and do occur simultaneously, but for clarity, we now describe and illustrate each individually. Note that impairment per se is not sufficient to justify a determination of lack of DMC. In the following section, we describe two further conditions that must also be met for someone to be judged unable to make their own decisions on the basis of impairment to the ability to value.

### 4.1 Impaired value possession

Imagine a severely depressed patient who must make a decision about treatment, but does not care whether they live or die. They have no desire to continue living, and they have no desire to expedite dying. A clinician might judge that they satisfy the criteria of the four abilities model: they exhibit understanding, appreciation, reasoning about their situation, and can communicate a choice (by refusing the recommended treatment). Nevertheless, the clinician might think that something is awry. Suppose, during the assessment, the patient also reports they do not care what is done. The refusal is simply a “preference” toward inaction as there is no reason to do anything; there is only apathy toward the decision itself. Here, the ability to value is impaired because there are no values—internal reasons for action—to engage within the decision‐making process.

### 4.2 Impaired access to values

An alternative possibility regarding the severely depressed patient is that they do possess values, but the depression is impeding their access. In this case, access to values is impaired by a lack of affect. In other cases, strong emotions can themselves impede access to one's values.^16^ Consider someone whose only treatment option is by injection, but who greatly fears needles. During decision‐making, the fear completely overrides other relevant values, including a previously expressed preference to receive treatment. They understand why they *should* want to receive the injection, but they *do not feel like* they want it. While emotional states sometimes illuminate which values are relevant to a decision, those that block a person's access to pertinent values are a source of impairment.

### 4.3 Impaired practical reasoning

The third possibility for impairment has to do with various ways in which practical reasoning can misfire. For example, one manifestation of impairment to practical reasoning occurs in cases of inner conflict which leads to ambivalence in decision‐making. Take the case of a patient with schizophrenia who agrees to have an operation for a pacemaker placement when speaking with a reassuring senior cardiologist, but over the course of 2 days repeatedly refuses the pacemaker when a young cardiology fellow tries to take him to the operating room. They repeatedly go back and forth between deciding to have the operation and refusing to have the operation. An inability to practially reason with their values, due to ambivalence caused by emotional states, leads to an impairment to their ability to value. Their decision is dictated by their mood and emotions of the moment and is not stable enough to allow a decision that can be implemented.^17^

### 4.4 Impaired action

The fourth type of impairment has to do with acting in accordance with the conclusion of practical reasoning. An example of this type of impairment is a patient with addiction who succumbs to a pathological impulse to use substances. They might possess, access, and reason with their values to the conclusion not to use substances, and then succumb to the impulse to use at the moment of action. Succumbing to impulse thwarts the ability to act in accordance with the valuing process and constitutes impairment to the ability to value.

## 5 JUSTIFYING A LACK OF CAPACITY ON THE BASIS OF IMPAIRMENT TO THE ABILITY TO VALUE

We have argued that the ability to value is necessary for someone to possess DMC, and that this ability has four parts that can be impaired. Under what conditions should someone be deemed to have an impairment in their ability to value such that they lack DMC? Here, we argue that someone should be deemed to lack DMC only if they meet both of the following conditions: the impairment to their ability to value is substantial and the impairment is due to some impairment in the functioning of the brain or mind.

The first condition follows from the fact that most adults are competent to make their own medical decisions and yet no one is perfect in their exercise of the abilities underlying DMC.^18^ For example, most of us are bad at understanding and applying information about risk; yet, our DMC is not called into question every time we are called upon to make a decision under conditions of uncertainty. Although competence is a categorical concept, its determination relies on phenomena that are matters of degree. In a given decisional context, over a certain threshold of abilities, an individual is regarded as having the right to make their own decisions. This means that a judgment that someone lacks DMC is justified only if their ability to understand, appreciate, or reason with the relevant information is substantially impaired, such that they do not reach that threshold. We do not take a position on the location of the threshold.

The same point applies to the ability to value. Our existing values don't cover every situation in which we find ourselves, they sometimes conflict so that we don't know what to do, and we sometimes make decisions which are mistaken even by our own lights. This is all part of being a human engaged in practical reasoning. For example, most people with addictions who succumb to impulse, acting self‐destructively and against their better judgment are still judged to be competent decision‐makers. They are considered to meet the threshold for DMC, despite making decisions that are mistaken by their own lights. The judgment that an impairment to the ability to value is substantial enough to entail a lack of DMC will follow the same approach as judgments of impairment to any area of capacity. The degree of impairment relevant to a specific medical decision will be evaluated at the time of the assessment, and we leave calculations of substantiality to the assessors’ experience and judgment. Our main point here is that just because some impairment of the ability to value can be identified does not imply automatically that a person lacks DMC, just as a person can lack some understanding and yet have DMC.

The second condition is more an epistemic safeguard than a justificatory criterion: the impairment must be due to or caused by a dysfunction or pathology of the mind or brain. This need not necessarily mean a diagnosis but the impairment should be explainable by current standards of clinical science as some form of dysfunction. Apparent deficits—even substantial ones—to someone's ability to value that are not traceable to or explainable by some dysfunction of mind or brain should not be taken as evidence that someone lacks DMC. Without this condition, there is too high a risk that a clinician would regard decisions made on the basis of values that were unusual or of which they disapproved as indicative of a lack of DMC. Clinicians and others who carry out capacity assessments should not be in the business of evaluating the content of patients’ values.^19^ Standards to justify a lack of DMC should be rigorous enough to accommodate decisions based on unusual beliefs or “bad decisions,” but because pathological causes are particularly challenging for patients to overcome, we should take seriously the effect pathology can have on capacity and autonomous decision‐making.

We emphasize here that the mere presence of a diagnosed psychiatric condition *does not* entail lack of capacity.^20^ Many people with mental illness—including patients with schizophrenia, severe depression, and other serious conditions—retain DMC, and “normal” people may have impairment in the functioning of mind or brain that could be sufficient to make them unable to make a decision. A reasonable causal explanation is only a necessary requirement.

## 6 THE ABILITY TO VALUE AND CHALLENGING CASES

In this section, we show how our conception of the ability to value can explain some persisting questions about capacity in challenging cases.

Patients with anorexia nervosa who refuse to eat can present challenges for judgments of DMC. Such patients frequently pass clinical assessments of DMC, such as the MacCAT‐T.^21^ Nonetheless, the decision to refuse food and treatment to the point of risking one's life raises the question of impairment. Some commentators have suggested that a preference for thinness to the point of bodily harm represents a pathological value. However, judging that someone lacks DMC on the grounds of the content of their values violates *value neutrality*—it is not for clinicians to decide which values are correct. Other analyses expand the appreciation criterion to include some role for values,^22^ or suggest distinguishing authentic from inauthentic values.^23^ We think that considering these patients in the light of the ability to value can help making principled judgments about their DMC.

Many patients with anorexia report conflicting values. Someone may place a very high value on being thin while also not wanting to die or experience the extreme physical side‐effects of starvation.^24^ Of course, there is nothing unusual about finding that one's values conflict—most people facing a tough decision find that they have some values that point one way and some that point another. They must then decide which values are more important. Some patients struggle to resolve this conflict. Hope et al. report that many of the patients they interviewed had two conflicting mindsets, suggesting “that people with anorexia nervosa experience not only frequent changes in preferences and beliefs but may be profoundly ambivalent at any one time.”^25^ One participant said:

But again it's the two headed thing one part of you says, like, you need some help… but the other part of you is screaming at you to run 600 miles in the opposite direction… you're caught between a rock and a hard place. [P39]^26^

In these cases, the patients with anorexia are able to access values, but their ability to engage in practical reasoning with those values is impaired. In other cases, a patient's emotions seem to be driving their decisions in contradiction to their values. For example, they may judge that it would be better overall to receive treatment and yet express their refusal. Again, this seems like a problem with practical reasoning.

Another version of the case involves a problem with decision‐making whereby the patient with anorexia refuses treatment but simultaneously expresses a desire to accept treatment. In this case, the patient explains that they wish they could force themselves to eat, but they just can't bring themselves to do it. They know that not eating will likely kill them, but they feel powerless to eat. They might be able to identify all of the relevant values, apply their values to their situation, and then be unable to bring themselves to say yes to food (or life‐saving treatment):

When I was lying in the hospital my mum would bring over a bowl of melon and say “Melon OK it's just water” …I'd thinking, “oh I really want to eat that, I want to eat, I just want to eat.” … And then when it came to it, my hands would start shaking and I'd just want to throw it across the room, I just couldn't do it, no matter how hard I tried I just couldn't physically do it … I don't even know what was stopping me, it was obviously the anorexia but my thought just changed, like one minute I would, and the next minute I just went “NO” I couldn't do it, at all. [P13]^27^

In this case, the patient decides they want to eat, but they can't execute their decision. This is an impairment to the action component of the ability to value.

Whether or not a patient with anorexia who is refusing treatment has an impairment to their ability to value will require extended discussion with the patient about their decision‐making. Given the variety of ways in which the condition manifests, no blanket determination can be made. Likewise, as with other impairments to the abilities that underlie DMC, it is a further question whether an impairment to someone's ability to value is sufficiently severe to render them not competent to make their own treatment decisions. Finally, even if someone lacks DMC, it does not follow that treatment should be forced on them against their will. What should be done depends on what is in the interests of the patient. Whether and when coercive measures are beneficial in the long run for patients with anorexia will likely vary by case.^28^

One advantage of focusing on the ability to value rather than the content of values themselves is the flexibility to account for unusual values. Take the case of a person with body integrity identity disorder (BIID) who desires to amputate a healthy limb: “The patient feels that the limb is not his, not part of him, and alien, causing distress. The pathology here is the feeling of alienation toward one's own limb.”^29^ Notwithstanding the unusual content of the desire, the patient reasons with their values—comparing a future of distress against a future without the particular limb—and decides to amputate. When describing their valuing process, the patient expresses that they value a life without the constant feeling of distress about their limb. They prioritize relief from their distress above life with the limb, and they decide to pursue amputation. In the case of BIID, we also happen to know that amputation does in some instances provide relief and improve well‐being.^30^ Our BIID patient is able to identify relevant values, apply their values to the decision at hand, and act accordingly. Despite the presence of pathology, there appears to be no impairment to the ability itself, and therefore no justification for judging a lack of capacity on the basis of impairment in the ability to value. Moving from assessing the values themselves to assessing the ability to value reduces the risk that a clinician could determine a lack of capacity based on disagreement about the content of the values. For our BIID patient, clinicians might not agree with the patient's values or their decision, but the patient demonstrates an intact ability.

## 7 OBJECTIONS

One objection to our proposal relates to our reliance on the notion of “impaired functioning of brain or mind” as a necessary condition for determining someone's ability to value is so impaired that they lack DMC. One might worry that we are saying only persons with psychiatric disabilities are open to being determined incompetent on this ground. This is not our view. We do not propose that *diagnoses* are necessary, only that a reasonable case be made that a person's “mind or brain” is in some explainable way not functioning as it should. Persons without psychiatric diagnoses could very well have episodes of such dysfunction sufficient to make them incompetent. The requirement is included as an epistemic safeguard, without which there would likely be more cases in which people with merely “unusual” values were judged incompetent. We think that this requirement will likely lead to assessments that are more objective and less prone to such abuse.

Second, someone might question the practical utility of adding the ability to value criterion. Two related factors make identifying impairments to the ability to value particularly complicated. One is that it is hard to reliably ascertain what someone's motivations truly are. We have defined a value in terms of the reason or justification for a decision, but neither the DMC evaluator nor even the decision‐maker may have direct access to the values that led to a decision. One can be mistaken about one's own motives. This difficulty is compounded by the fact that the abilities that comprise DMC are interdependent and interact with each in ways that affect what we observe. For example, the abilities (and components within an ability) can be hierarchical. So the ability to intellectually grasp and comprehend information is a necessary prior step, in order for the person to then apply that information to their situation (i.e., one must understand in order to appreciate). Thus, on evaluation, loss of appreciation will be seen when there is lack of comprehension as well as in primary deficits of appreciation. Likewise with “retaining information” (an ability specified in MCA 2005), which is a necessary precondition for being able to use or weigh that information.^31^

These two factors can complicate how we detect impairments in the ability to value. Impairments in the ability to value can manifest in multiple domains simultaneously. It may be in practice very difficult to distinguish between someone who lacks a relevant value and someone who fails to access that value. A patient who lacks relevant values—for example, someone who is completely apathetic—may also appear to lack the ability to reason with or act in accordance with values. Or take the patient who is experiencing an overwhelming emotional state and is unable to identify their values—they will likely not seem to be able to practically reason, either. This explains why slight changes in scenarios depicting impaired persons can seemingly change the explanations for why a person lacks DMC in such a situation.

The practical consequence of all this is that the ability to value as a component of DMC should not be thought of as something that can be “ticked off” a checklist in an evaluation. Rather, it should be thought of as a criterion that needs to be kept in mind and probed on those occasions when the more “upstream” and cognitive components of DMC appear to be intact, but when the person's decision‐making still seems influenced by some dysfunction of mind or brain. Given the complexity of relationships between the abilities, it may not be possible to specify ahead of time a list of manifestations that phenomenologically exhaust the ways a person's ability to value can go wrong in a decision‐making situation.

A third objection raises a related but distinct practical objection. In response to various proposals to revise the four abilities model, Paul Appelbaum has argued that the four abilities model can deal with many apparently challenging cases and that there should be a high burden of proof for adding any additional criterion for assessing DMC.^32^ For any proposed new criterion, we should ask about: “Its sensitivity in correctly identifying people with impairment, its specificity in avoiding labeling unimpaired people as incapable, and the reliability with which it can be applied (i.e., whether different assessors are likely to come to the same conclusion about the same person).”^33^ According to Appelbaum, even if the ability to value is truly a component of DMC, this does not mean that we should add a corresponding criterion to the tools used to assess DMC. Doing so might make actual practice even more prone to error.

In response, we think that the four abilities model can only deal with the challenging cases we have discussed if the appreciation criterion is expanded so far as to effectively include the ability to value. As currently applied, it therefore lacks sensitivity. However, we acknowledge that concerns about the sensitivity, specificity, and reliability of possible measures of the ability to value are valid. We have not proposed specific amendments to existing instruments for assessing DMC and so we lack data on how such amended instruments would perform in practice. Were incorporation of the ability to value into capacity assessments to make things worse, that would be reason to reconsider. Yet the absence of such data is not a reason to retain the status quo. Rather, we should proceed with caution and collect data on the success or failure of alternative criteria for assessing DMC.

## 8 CONCLUSION

In medicine, the model of decision‐making capacity exists to protect and preserve the autonomy of individuals. It seems intuitive to expect that the model for DMC should map onto the decision‐making process itself. If so, we must be careful that models are not too narrow, so that they exclude the role of emotions and values in decision‐making. A complete model for capacity must include the role of the ability to value, since values are the motivating force behind decisions. Adding the ability to value as an additional criterion for DMC can fill in gaps in those models that are too narrowly cognitive and provide structure to those models that appear to go beyond the cognitive (such as the MCA's “use or weigh” criterion). Many impairments to DMC can be explained as impairments to one or more of the components of the standard four criteria model. But not all. Those engaged in capacity assessments should be alert to the possibility of impairments to the ability to value. Such impairments can occur alongside other impairments or along and, when certain conditions are met, can entail a loss of DMC.

## Footnotes

1 In addition to the authors cited in Sections 2 and 6, see Charland, L. C. (1998). Appreciation and emotion: Theoretical reflections on the MacArthur Treatment Competency Study. *Kennedy Institute of Ethics Journal*, *8*(4), 359–377; Charland, L. C. (2006). Anorexia and the MacCAT‐T Test for mental competence: Validity, value, emotion. *Philosophy, Psychiatry, Psychology*, *13*(4), 283–287; Elliot, C. (1997). Caring about risks: Are severely depressed patients competent to consent to research? *Archives of General Psychiatry*, *54*(22), 113–116; Hermann, H., Trachsel, M., & Biller‐Andorno, N. (2017). Accounting for intuition in decision‐making capacity: Rethinking the reasoning standard? *Philosophy, Psychiatry, & Psychology*, *24*(4), 313–324.

2 Appelbaum, P. S. (2007). Assessment of patients’ competence to consent to treatment. *New England Journal of Medicine*, *357*(1836), 1834–1840.

3 National Health Service. Mental Capacity Act. (2021). https://www.nhs.uk/conditions/social-care-and-support-guide/making-decisions-for-someone-else/mental-capacity-act/

4 Tan, J., Stewart, A., Fitzpatrick, R., & Hope, R.A. (2006). Studying penguins to understand birds. *Philosophy, Psychiatry, & Psychology,13*(4), 299.

5 Tan, J., Hope, T., & Stewart, A. (2003). Competence to refuse treatment in anorexia nervosa. *International Journal of Law and Psychiatry*, *26*(6), 697–707.

6 Hope, T., Tan, J., Stewart, A., & McMillan, J. (2013). Agency, ambivalence and authenticity: The many ways in which anorexia nervosa can affect autonomy. *International Journal of Law in Context*, *9*(1), 20–36.

7 Halpern, J. (2012). When concretized emotion‐belief complexes derail decision‐making capacity. *Bioethics*, *26*(2), 108–116. Halpern also says this can be seen as a failure in appreciation, where the standard is conceived more broadly than what she considers a too narrow a framework: “Currently, psychiatrists apply the appreciation criteria narrowly, looking at how the person understands the facts about medical treatment and her medical situation rather than at how the person views the impact of treatment on her overall quality of life” (110).

8 Hermann, H., Trachsel, M., Elger, B. S., & Biller‐Andorno, N. (2016). Emotion and value in the evaluation of medical decision‐making capacity: A narrative review of arguments. *Frontiers in Psychology*, *7*(765), 1–10.

9 Hawkins, J. (2023). Affect, values and problems assessing decision‐making capacity. *The American Journal of Bioethics*, *24*(8), 1–12.

10 Ibid.: 7. The authors of this paper disagree about whether Hawkins’ view overlaps considerably with our own (such that the two make similar proposals but using different terminology) or whether her view and ours are rivals (such that a decision must be made about whether to ground DMC in welfare or in a conception of autonomy that does not reduce to welfare considerations).

11 The National Commission for the Protection of Human Subjects of Biomedical and Behavioral Research. (1978). *The Belmont report: Ethical principles and guidelines for the protection of human subjects of research*. US Department of Health, Education, and Welfare.

12 See, for example, Dworkin, R. (1993). *Life's dominion* (pp. 222–226). Alfred A. Knopf; Jaworska, A. (1999). Respecting the margins of agency: Alzheimer's patients and the capacity to value. *Philosophy & Public Affairs*, *28*(2), 105–138.

13 One example comes from Brock and Buchanan: “a competent decision‐maker also requires a set of values or conception of what is good that is at least minimally consistent, stable, and affirmed as his or her own.” Buchanan, A., & Brock, D. (1990). *Deciding for others* (p. 25). Cambridge.

14 Cf. Jaworska, op. cit., note 12, p. 116.

15 Kim, S. Y. H, Kane, N. B., Ruck, A. K, & Owen, G. S. (2022). Broad concepts and messy realities: Optimising the application of mental capacity criteria. *Journal of Medical Ethics*, *48*, 838–844.

16 Kane, N. B., Keene, A. R., Owen, G. S., & Kim, S. Y. H. (2021). Applying decision‐making capacity criteria in practice: A content analysis of court judgments. *PLoS ONE*, *16*(2), e0246521.

17 Note that ambivalence in itself does not entail an impairment to the ability to value. One may be ambivalent about a decision because of genuine uncertainty about what is the right thing to do. Such ambivalence may persist despite impeccable practical reasoning. That is quite different from a situation in which one vaccilates between strongly held views depending on one's emotional state.

18 Cf. Hawkins’ “inclusivity” constraint: “When building a model for assessing capacity, one must be careful not to build in too much, since then one might arrive at a model according to which too many people lack capacity” (Hawkins, op. cit., note 9, p. 5).

19 This is Hawkins’ “value neutrality” constraint (ibid: 4). Cf. Kim, S. Y. H. (2016). The place of ability to value in the evaluation of decision‐making capacity. In D. Moseley & G. Gala (Eds.), *Philosophy and psychiatry: Problems, intersections and new perspectives* (p. 193). Routledge.

20 Hawkins’ “diagnostic neutrality” constraint (ibid: 4–5).

21 Tan, J., Hope, T., Stewart, A., & Fitzpatrick, R. (2006). Competence to make treatment decisions in anorexia nervosa: Thinking processes and values. *Philosophy, Psychiatry, & Psychology: PPP*, *13(*4), 267.

22 Grisso, T. & Appelbaum, P. S. (2006). Appreciating anorexia: decisional capacity and the role of values. *Philosophy, Psychiatry, & Psychology*, *13*(4), 293–297.

23 Hope, T., Tan, J., Stewart, A., & Fitzpatrick, R. (2011). Anorexia nervosa and the language of authenticity. *Hastings Center Report*, *41*(6), 19–29.

24 Ibid: 22.

25 Hope, T., et al., op. cit. note 6, p. 31.

26 Ibid: 31.

27 ibid: 25.

28 Schreyer, C. C., Coughlin, J. W., Makhzoumi, S. H., Redgrave, G. W., Hansen, J. L., & Guarda, A. S. (2016). Perceived coercion in inpatients with Anorexia nervosa: Associations with illness severity and hospital course. *International Journal of Eating Disorders, 49*(4), 407–412; Ward, H., Ramsay, R., Russell, G., & Treasure, J. (2015). Follow‐up mortality study of compulsory treated patients with anorexia nervosa. *International Journal of Eating Disorders, 48*(7), 860–865.

29 Kim, op. cit., note 21, p. 200.

30 Ibid.

31 Kim, et al., op. cit. note 15.

32 See, for example, Appelbaum, Paul, S. (2017) Intuition, self‐reflection, and individual choice: Considerations for proposed changes to criteria for decisional capacity. *Philosophy, Psychiatry, & Psychology*, *24*(4), 325–328; Appelbaum, P. S. (1998) Ought we to require emotional capacity as part of decisional competence? *Kennedy Institute of Ethics Journal*, *8*(4), 377–387; Trachsel, M., & Appelbaum, P. S. (2020) A narrative coherence standard for the evaluation of decisional capacity: Turning back the clock. *AJOB Neuroscience*, *11*(1), 1–3.

33 Ibid. (2017): 327.

